# Treatment of Chagas Disease in the United States

**DOI:** 10.1007/s40506-018-0170-z

**Published:** 2018-06-26

**Authors:** Sheba Meymandi, Salvador Hernandez, Sandy Park, Daniel R. Sanchez, Colin Forsyth

**Affiliations:** Center of Excellence for Chagas Disease at Olive View-UCLA Medical Center, 14445 Olive View Drive, Sylmar, CA 91342 USA

**Keywords:** Chagas disease, *Trypanosoma cruzi*, Neglected tropical diseases, Benznidazole, Nifurtimox

## Abstract

**Purpose of Review:**

Chagas disease (CD) is endemic to much of Latin America, but also present in the United States (U.S.). Following a lengthy asymptomatic period, CD produces serious cardiac or gastrointestinal complications in 30–40% of people. Less than 1% of the estimated six million cases in the Americas, including 326,000–347,000 in the U.S., are diagnosed. Infected persons are typically unaware and the bulk of clinicians are unfamiliar with current treatment guidelines. This review provides U.S. and other clinicians with the latest knowledge of CD treatment.

**Recent Findings:**

Chagas cardiomyopathy (CCM) causes severe fibrosis and autonomic damage in the myocardium. Eliminating the parasite through antitrypanosomal therapy with benznidazole, a nitroimidazole derivative or nifurtimox, a nitrofuran compound, potentially prevents heart failure and other sequelae of advanced CCM. Benznidazole, recently approved by the U.S. Food and Drug Administration (FDA) for children 2–12 years old, is the first-line therapy; optimal dosages are currently being studied. Antitrypanosomal therapy prevents congenital transmission; produces high cure rates for acute, congenital, and early chronic cases; and improves clinical outcomes in adult chronic indeterminate cases. However, this benefit was not observed in a large clinical trial that included patients with advanced CCM.

**Summary:**

Treatment with antitrypanosomal drugs can cure CD in acute, congenital, and early chronic cases and provides improved clinical outcomes for chronic indeterminate cases. This treatment should be offered as early as possible, before advanced CCM develops.

## Introduction

Over six million people worldwide are infected with *Trypanosoma cruzi*, the flagellate protozoan which causes American trypanosomiasis or Chagas disease (CD) [1–3], but the overwhelming majority are undiagnosed. In most cases, CD is transmitted by hematophagous insects of the subfamily *Triatominae*, known as kissing bugs in the United States (U.S.) and by many regional names in Latin America, mainly *chinches picudas*, *vinchucas*, *pitos*, *barbeiros*, *chipos*, *and chinchorros*, *among others*. Triatomines capable of transmitting the parasite are widespread in the Americas, ranging from the southern half of the United States to Argentina and Chile. Certain species became domiciliated in Latin America, in particular favoring houses made of mud, thatch, and adobe which provide abundant hiding and nesting places. Consequently, CD has typically been concentrated among the rural poor of Latin America, though vector transmission in urban areas and the U.S. is also well documented [4, 5]. *T. cruzi* has both sylvatic and domestic transmission cycles. Several species of mammals, including dogs, cats, opossums, armadillos, raccoons, and wood rats, serve as reservoirs. The parasite inhabits the gut of the triatomine. Upon feeding, the triatomine defecates, and the sleeping host unwittingly introduces the infected feces into the bloodstream by scratching the site of the bite. CD can also be transmitted through consumption of food contaminated by triatomine feces, blood transfusion and organ transplant. Moreover, CD can reactivate in cases of immunosuppression. Additionally, the congenital transmission rate in infants born to CD-positive mothers ranges from 2 to 5%, with higher rates for births in endemic settings [6, 7].

CD is characterized by acute and chronic phases. The acute phase typically begins 1–2 weeks after infection; symptoms are generally absent or non-specific, similar to a viral illness. Therefore, the acute phase is almost always unrecognized. However, it can occasionally cause fatal myocarditis or meningoencephalitis. During the acute phase, *T. cruzi* trypomastigotes can be observed swimming freely in the bloodstream.

Following the acute phase, the trypomastigotes transform into amastigotes to hide from the immune response and lodge in deep organ tissue, particularly the heart and digestive tract. The infection enters a chronic indeterminate phase, which, unless antiparasitic treatment is administered, will endure the lifespan of the patient. While 60–70% of patients remain asymptomatic, the remainder will progress to an advanced chronic phase, usually 10–30 years after the initial infection [8, 9]. This progression is almost certainly triggered by parasite persistence, which probably acts in concert with tissue damage caused by the immune response [10, 11].

*T. cruzi* exhibits substantial genetic diversity and has been classified into six discrete typing units [12]. This diversity, in combination with other mechanisms including host immune response, may contribute to variability in both the clinical manifestations of CD and responses to antiparasitic treatment [13]. Chronic symptoms are usually cardiac-related, especially when the infection is acquired in North America. Cardiac manifestations fall into four principal categories: progressive heart failure, cardiac arrhythmia, conduction abnormalities, and thromboembolism [9]. Ventricular tachycardia, both sustained and non-sustained, is frequently observed. Abnormalities in the conduction system, particularly, but not limited to, right bundle branch block and left anterior fascicular block, are characteristic of chronic CD and may serve as early warning signs of clinical progression [9]. Chagas heart disease is frequently fatal with sudden death causing roughly two thirds of mortality [8].

Digestive manifestations are also possible and account for a third of chronic cases in the Southern Cone of South America. The digestive form most often presents as megaesophagus or megacolon, the latter with severe constipation and abdominal distension. Rarely, CD impacts the nervous system, producing nodular encephalitis, peripheral neuropathy, and cerebral masses in immunocompromised patients [14].

## Public health importance

With > 99% of an estimated 6 million CD patients undiagnosed in the U.S. and Latin America, there is a need for more comprehensive screening and incorporation of diagnosis and treatment into the primary care setting. Annually, CD causes over 7000 deaths and a considerable burden in morbidity, more than any other parasitic disease in the Americas [15]. Global annual healthcare costs from CD were estimated at US$627.46 million in 2013 (equivalent to $685.52 million in 2018); Brazil and the United States ranked first and second for annual expenditures [16]. A recent European study indicates screening of Latin American-born patients in primary care would be a highly cost-effective measure [17]. Such screening has yet to be systematically implemented in the United States [18•].

Both provider and patient awareness of CD are extremely low, creating substantial barriers to diagnosis and treatment. In a 2010 survey, 57% of a sample of 1142 U.S. clinicians either had not heard of CD or felt little confidence that their knowledge was up to date [19]. When widespread screening of the blood supply in the U.S. began in 2007, a systematic review describing treatment options for U.S. clinicians was published [20]. Since that time, new international guidelines and the results of several clinical studies have been published.

The purpose of the present article is to familiarize clinicians with the most recent guidelines and therapeutic advances in CD and to share the experience and insights of the Center of Excellence for Chagas Disease (CECD) at Olive View-UCLA Medical Center in Los Angeles, one of the few U.S. providers currently offering treatment for CD.

## Screening and diagnosis

U.S. clinicians should screen patients who were born in Latin America, who have spent > 6 months in a rural area of Latin America, and/or who report exposure to triatomines. In a study of Latin American-born patients in Los Angeles, having lived in housing made of mud, adobe, and thatch, having a family member with CD, and Salvadoran origin were predictors of *T. cruzi* infection [21]. In the acute phase, CD can be diagnosed through direct observation of *T. cruzi* in peripheral blood. However, patients will typically need testing in the chronic phase when detection of the parasite is more difficult. Clinical diagnosis relies on positive serology on a minimum of two tests with different antigenic principles [22]. Various assays with a range of performance characteristics are commercially available [23]. For chronic cases, recent Brazilian guidelines recommend using a test with high sensitivity such as a total-antigen enzyme-linked immunosorbent assay (ELISA) or indirect immunofluorescence assay as an initial test, followed by a highly specific method such as an indirect hemagglutination assay [24]. If the two tests are discordant, a Western blot may be used as a tiebreaker.

**Fig. 1:**
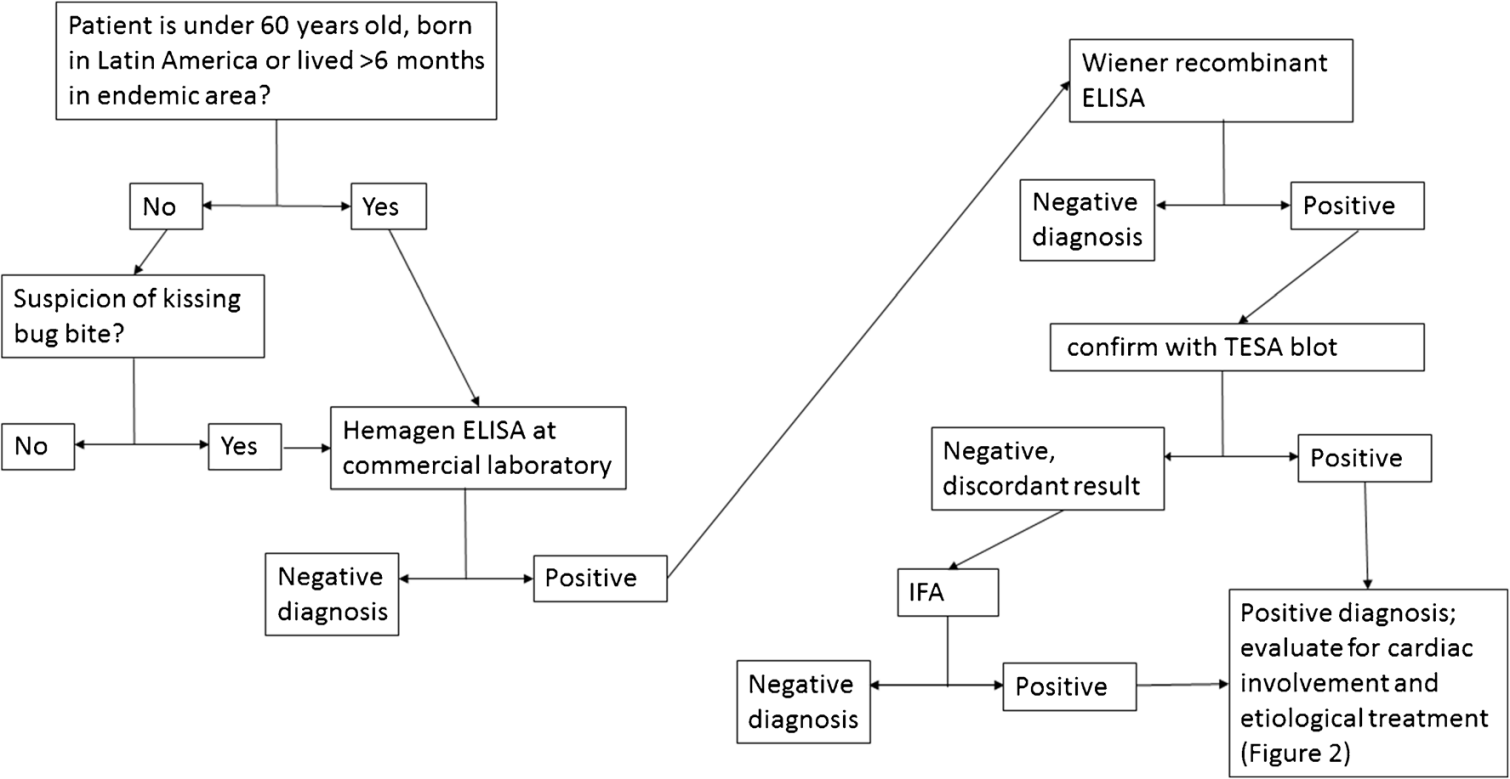
Diagnostic process of the center of excellence for Chagas disease at olive view-UCLA medical center. Patients eligible for treatment are first tested at a commercial laboratory. If that result is positive, patients receive further testing (a Wiener ELISA and TESA blot) at the CDC.

In the U.S., four commercial immunoassays for clinical use have some level of FDA approval: three ELISAs (Wiener Chagatest ELISA recombinante, Hemagen ELISA, and Ortho *T. cruzi* ELISA) and one rapid assay (InBios Chagas Detect Plus). Figure 1 illustrates the diagnostic process used by the CECD in Los Angeles, which has screened over 8000 people in the U.S. for *T. cruzi* since 2007. Assays have shown varying performance characteristics in different settings and patient populations [25], which may be due to *T. cruzi* genetic diversity [26] and/or geographically patterned variations in immune responses [27]. This is an especially critical point in light of the heterogeneous nature of the Latin American-origin population in the U.S. A World Health Organization comparative evaluation provides performance characteristics for several assays based on a multinational panel [23], while a recent meta-analysis includes summary estimates of sensitivity and specificity for both the Wiener Chagatest ELISA (93.7 and 99.0%, respectively) and the Ortho *T. cruzi* ELISA (99.2 and 99.1%, respectively). However, the authors caution that a tendency to use only well-defined positive or negative samples in prior research may fuel overestimation of sensitivity and specificity [28]. How assays perform in a particular clinical setting may vary significantly, underscoring the importance of using at least two assays based on different antigenic principles to diagnose *T. cruzi* infection.

## Antitrypanosomal therapy

### New developments

Benznidazole, a nitroimidazole derivative (N-Benzil 2 Nitro 1-Imidazolacetamide), and nifurtimox, a nitrofuran compound, both developed over 40 years ago, are currently the only drugs available for treating CD.

Benznidazole is often considered the first-line therapy because of its better tolerability, but both drugs produce significant side effects. These nitroheterocyclic drugs inhibit the parasite’s ability to replicate DNA, and are effective against the trypomastigote and amastigote forms [29]. Effectiveness is higher for both drugs if administered as soon as possible after infection**.** Reported cure rates are as high as 96% for congenitally infected infants [30], 76% for acute infections [31], 62% for chronically infected children [32], and 37% for chronically infected adults [33]. Efficacy appears to decrease in proportion to the length of the infection, although treatment success is difficult to measure with current diagnostic tools. It can take years subsequent to treatment before patients with chronic CD become seronegative (serorevert). In one study, the average time to serorevert in 20 successfully treated chronically infected adults was 16 years [33]. Tools relying on parasite DNA detection, principally polymerase chain reaction, are impractical for use in primary healthcare settings and unreliable for verifying cure as results are variable, although parasite DNA detection following treatment is a clear indication of therapeutic failure.

In part due to limited evidence of efficacy, and partly because of increasing frequency and severity of side effects in relation to patient age, treatment decisions have historically hinged on age categories. Evidence in favor of treating congenital cases, acute cases, and children in the early chronic phase is well established [30, 34, 35]. However, the BENEFIT trial, a multinational randomized study comparing benznidazole and placebo in patients who already had developed advanced CCM, did not find significantly different outcomes [36••]. This makes it essential to initiate antiparasitic treatment as early as possible in acute or indeterminate patients, before the onset of more severe forms of CCM.

It was previously believed that chronic manifestations were due primarily to an overactive immune response and that antiparasitic treatment would therefore be futile in adult patients in the chronic phase. However, long-term studies in Latin America have demonstrated better clinical outcomes for adult patients with chronic CD treated with benznidazole or nifurtimox [33, 37], which complements the latest understanding of CD pathology as at least partly triggered by parasite persistence [11]. Recent clinical trials demonstrated benznidazole is highly efficacious at clearing the parasite, as measured by repeatedly negative PCR, in 65–87% of adult patients in the chronic indeterminate phase of the disease [36••, 38–40], although a smaller percentage revert to negative serology. Expert consensus now favors making treatment available to adults in the chronic indeterminate phase up to age 50. Antiparasitic treatment likely prevents or delays severe complications from chronic CD. For women of childbearing age, antiparasitic treatment has been proven to eliminate the possibility of congenital transmission [41–43].

### Recommendations and contraindications

Current treatment recommendations take into account the phase of the disease and age of the patient (Table 1). Acute and congenital cases, reactivations, and children in the chronic indeterminate phase should be offered antiparasitic treatment with benznidazole. Nifurtimox should be used in the event the patient is not able to tolerate benznidazole, or if benznidazole is unavailable within the health system. Adults in the indeterminate phase or with minimal cardiac involvement up to age 50 should be offered treatment. There is not sufficient evidence supporting effectiveness of treatment in older adults; however, treatment may be considered on a case-by-case basis for adults over 50 without contraindications or advanced cardiac involvement [24]. Even though a majority of patients in the indeterminate phase will remain asymptomatic, currently, there is no way to predict which patients will progress to the determinate form of the disease. Therefore, treating all patients without contraindications remains the best means of preventing CD-related mortality and morbidity.

**Table 1:** Recommendations for antiparasitic treatment of *T. cruzi* infection

Clinical group	Treatment recommendation [44]	Recommendation, evidence level [24]
Infants with congenital infection	Treat	I, B
Any acute phase	Treat	I, B
Reactivation in immunocompromised	Treat	I, C
Children in chronic indeterminate phase	Treat	I, A
Adolescents in chronic indeterminate phase	Treat	IIa, B
Seropositive organ donors	Treat	I, C
Recipients of organs from seropositive donors	Probable treat	IIa, C
Laboratory accidents	Treat	IIa, C
Women of childbearing age	Treat	NA
Chronic indeterminate phase, adults 19–50 years old without cardiomyopathy (Kuschnir 0)	Probable treat	IIa, B
Chronic phase, cardiomyopathy without advanced heart disease (Kuschnir I, II)	Probable treat	IIb, C
Chronic phase with advanced cardiomyopathy (Kuschnir III)	Probable non treat	III, C
Chronic indeterminate phase, adults older than 50 without advanced cardiomyopathy (Kuschnir 0, I, II)	Possible treat; case-by-case evaluation	IIb, C [20]
Early digestive involvement without advanced cardiomyopathy (Kuschnir 0, I, II)	Probable treat	IIa, C
Pregnant women	Definite non-treat	III, C

Definitions of classes from the Brazilian consensus: I—conclusive evidence supporting treatment, II—conflicting evidence or views, IIa—evidence and consensus favors treatment, IIb—treatment considered optional due to lack of definitive supporting evidence and conflicting views, III—conclusive evidence or consensus that treatment is not effective. Definitions of evidence levels: A—data from multiple randomized trials or meta-analyses of randomized trials; B—data from only one randomized trial or several non-randomized observational studies; C—supported by consensus of expert opinion

Treatment with benznidazole and nifurtimox is contraindicated during pregnancy due to limited evidence on safety. Although the current Brazilian consensus guidelines do not recommend treatment during breastfeeding [24], it is not contraindicated in the Argentinian guidelines [44] as some research indicates absorption of benznidazole or nifurtimox through breastmilk does not pose a risk to infants [45, 46]. Other contraindications are renal or hepatic insufficiency and moderate to severe cardiac dysfunction. Because of impaired absorption, patients with difficulty swallowing due to severe megaesophagus may require corrective interventions prior to antiparasitic treatment [20].

**Fig. 2:**
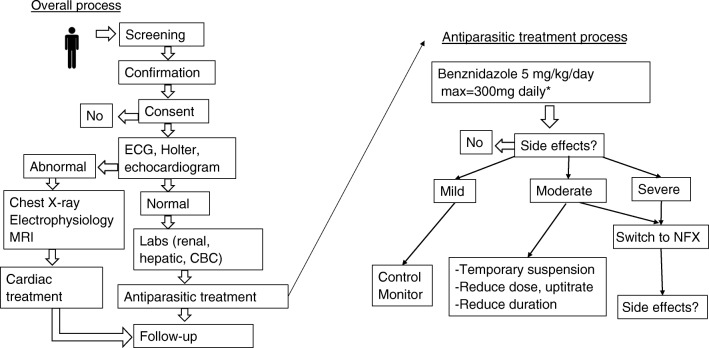
Clinical management of patients at the center of excellence for Chagas disease at Olive View-UCLA Medical Center. Patients are evaluated for chronic Chagas cardiomyopathy and undergo requisite labs (renal and hepatic function and complete blood count) to determine eligibility for etiological treatment. The right side of the figure illustrates management of side effects, which depends on their severity.

Figure 2 details the overall treatment process at the CECD. Prior to treatment, all patients should receive a complete blood count with differential, tests of renal and hepatic function and, for women of childbearing age, a pregnancy test. To gauge severity of cardiac involvement, patients should also receive at minimum an electrocardiogram and echocardiogram. A chest X-ray and 24-h Holter are also recommended in most international guidelines [24, 44, 47]. Kuschnir et al. developed a system of classifying severity of CD cardiomyopathy (Table 2) [48]. A prospective study found that adult patients in Kuschnir groups 0, I, and II who were treated with benznidazole had significantly lower risk of dying, progressing to a higher grade of severity, or developing new electrocardiographic abnormalities compared with untreated patients; the median length of follow-up was 9.8 years [37]. However, treatment is typically not indicated for patients with advanced heart failure (Kuschnir group III) based on the results of the BENEFIT trial [36**••**].

**Table 2:** The Kuschnir classification for chronic Chagas disease cardiomyopathy [48]

Kuschnir group	Serologic testing for *T. cruzi*	Abnormal ECG	Cardiac enlargement by chest X-ray	Clinical signs of heart failure
0	+	–	–	–
I	+	+	–	–
II	+	+	+	–
III	+	+	+	+

### Dosage

The recommended adult dosage for benznidazole is 5 mg/kg divided into two daily doses, not exceeding 300 mg in 1 day for 60 days. The recommended dose for children is 5-7 mg/kg daily divided into two doses. Benznidazole is ideally taken after meals to avoid gastrointestinal discomfort.

For nifurtimox, the adult dosage is 8–10 mg/kg daily divided into three daily doses and administered over 60 days. [The length of treatment was previously recommended as 90 days, but this has been reduced in recent international guidelines [24, 44].] Children should receive 10–15 mg/kg day divided into three daily doses.

### Treatment monitoring and side effects

Because both benznidazole and nifurtimox produce side effects in the majority of patients, biweekly monitoring with readministration of the baseline laboratory studies (complete blood count with differential, hepatic, and renal function testing) is recommended. While historically 20–25% of patients have discontinued treatment secondary to adverse effects (AEs), a program of close surveillance and intervention can reduce this rate considerably. The BENEFIT trial was able to reduce discontinuation secondary to AEs to only 13.4% of 1429 patients treated with benznidazole, despite an older cohort with a mean age of 55.4. (Morillo et al 2015) In a cohort of 2075 patients treated with benznidazole by Médecins sans Frontières/Doctors without Borders in Bolivia, in which patients were monitored weekly by clinical staff, only 10.2% discontinued therapy [28].

The most common AEs caused by benznidazole are skin disorders, which affect 26.3–52.9% of patients [49–53]. However, the majority of these reactions are mild and do not necessitate treatment interruption. While some observe that dermatological issues appear mainly in the first 2 weeks of treatment [29], other studies suggest these AEs can occur at any point [49]. Other reported side effects are digestive intolerance, anorexia, headache, and leukopenia. Dysesthesia is a potential outcome related to total accumulated dose which is occasionally observed as patients near the end of treatment. In a study of 30 patients treated with benznidazole at the CECD in Los Angeles, 47–50% experienced rash, headache, anorexia, and/or peripheral neuropathy [50]. However, subsequent reductions in the dosing regimen based on new international guidelines have resulted in an improvement in the side effect profile [54].

Nifurtimox produces more frequent and varied AEs than benznidazole, but the majority are mild. The most common AEs from nifurtimox are nausea, anorexia, abdominal pain, insomnia, headache, and amnesia [55–57]. In a CECD study of 53 patients treated with nifurtimox, there were frequent AEs (a mean of 8.2 affecting 100% of patients), but > 90% were mild and 79.2% of patients were able to complete treatment [57].

If a patient reports AEs, various actions can be taken. Many AEs are mild and will resolve spontaneously. For AEs of moderate severity, the dose can be reduced by 50% and uptitrated if patient tolerance improves. Alternatively, therapy can be temporarily discontinued and then reintroduced at a lower dosage. If a patient presents with a moderate to severe AE in the latter stages of treatment, discontinuation may be the best alternative. In the Argentinian guidelines, 30 days is deemed sufficient duration to consider treatment complete [44]. If a patient presents with a severe AE, immediate discontinuation is essential. In such cases, after a period of discontinuation and normalization of laboratory studies, nifurtimox may be offered as an alternative therapy. In a small sample of patients who discontinued benznidazole following hypersensitive reactions, nifurtimox was well tolerated [58].

Following treatment, negative serology is still the best way to ascertain a cure. Nonetheless, adults with chronic indeterminate CD may not have a negative serology until > 10 years after treatment. Care should be taken to explain to patients that a positive test result does not necessarily mean treatment has failed. After treatment, each patient should receive serological testing, an electrocardiogram, and an echocardiogram annually. Therapeutic failure should be concluded if there is evidence of clinical progression (e.g., new ECG abnormalities) even if CCM is still mild (Kuschnir I or II). In these cases, retreatment with a different trypanocide may be considered [24].

### Drug acquisition in the United States

Benznidazole was approved by the U.S. Food and Drug Administration (FDA) on August 29, 2017, for children ages 2–12, and may be prescribed off-label for adolescents, adults, and children under 2. Benznidazole is available through a central distributor and can be ordered at https://www.benznidazoletablets.com/en/. Nifurtimox has yet to receive FDA approval but is available in 120-mg tablets under the brand name Lampit through the Centers for Disease Control by way of the Pan American Health Organization, which in turn receives a donation of one million tablets annually from Bayer. Providers can only solicit nifurtimox from the CDC through a special investigational protocol; the drug is provided free of charge to patients.

## Treatment of chronic Chagas cardiomyopathy

CCM, which affects roughly 30% of patients infected with *T. cruzi*, typically engenders malignant ventricular arrhythmias, left ventricular dilation and dysfunction, apical aneurysms, congestive heart failure, and sudden death [8]. Malignant ventricular arrhythmias are more common in CD than other forms of heart disease and patients with CCM have a higher mortality than those with non-CD cardiomyopathy [59]. A study of Latin American-born immigrants with non-ischemic cardiomyopathy found 19% were positive for CD, and this group had a significantly increased risk of death or transplant (hazard ratio = 4.46) compared to those without CD [60]. A score was developed and validated by Rassi et al. to predict risk of death from CD heart disease [61].

Table 3 details the cardiac examinations all patients with confirmed *T. cruzi* infection should receive at baseline. Because antitrypanosomal therapy may be less effective once CCM is evident, patients with CCM should be referred to a cardiologist. Recent Brazilian guidelines address clinical management of CCM [24, 62], while ACC/AHA guidelines for heart failure [63] are applicable to CCM. Additionally, as described below, specific clinical features of CCM compared to non-CD cardiomyopathy deserve consideration.

**Table 3:** Recommended surveillance in patients with confirmed *T. cruzi* infection

Study	Comments	Frequency
Electrocardiogram	CD patients with a normal ECG have a good prognosis. The presence of conduction abnormalities may be an early marker of cardiac damage [9]. Right bundle branch block, often in tandem with left anterior fascicular block, is a hallmark of CCM. The number of abnormalities on ECG has been shown to correspond with the severity of myocardial damage [9].	Baseline and annually
Echocardiogram	Left ventricular systolic dysfunction triggered by fibrosis is a common feature of CCM. Wall motion abnormalities and apical aneurysms are frequent features of CCM.	Baseline and annually
24-h Holter	To evaluate the presence of arrhythmias and autonomic dysfunction, which elevate the risk of sudden death.	Baseline
Chest X-ray	To assess cardiomegaly.	Baseline

### Arrhythmias

Arrhythmias are one of the most frequent clinical outcomes of chronic determinate CD and lead to bradyarrhythmias, sick sinus syndrome, atrial fibrillation, ventricular tachycardia, and ventricular fibrillation. In Brazil, CD is responsible for 25% of all pacemaker implants [64]. In a study of Latin American-born patients with pacemakers in Los Angeles, 7.5% were found to have CD [65], suggesting CD as an underlying cause of cardiomyopathy may be severely underdiagnosed in the U.S. [66•].

A primary objective of treating CCM is preventing sudden death. An implanted cardioverter-defibrillator (ICD) is recommended for CD patients with sustained ventricular tachycardia, regardless of left ventricular ejection fraction (LVEF), and for CD patients who have recovered from cardiac arrest. Moreover, although data are limited, amiodarone may improve outcomes for patients at risk of sudden death due to non-sustained ventricular tachycardia with signs of myocardial dysfunction [8] and is recommended in Latin American guidelines [62]. An ongoing, randomized, prospective study in Brazil (CHAGASICS) is comparing mortality and hospitalization among patients treated with ICD or amiodarone [67].

### Use of ACE Inhibitors and Beta-blockers

Angiotensin-converting enzyme (ACE) inhibitors have been shown to reduce mortality in patients with CCM [68]. Bradycardia in CD patients could be aggravated by the use of beta-blockers [8]. Nonetheless, in a Brazilian randomized trial, beta-blocker therapy improved survival in patients with CCM compared to untreated patients [69]. Another randomized trial suggested carvedilol was safe and produced an increase in LVEF in patients with CCM [68]. In the latter study, ACE inhibitors were administered first and beta-blockers were given after patients’ clinical condition improved.

### Transplantation

Diagnosis of CD is not a contraindication for transplantation. In fact, a systematic review found that CD patients actually had better survival than non-CD patients following heart transplant [70]. CD-related heart failure is the third most common reason for heart transplantation in South America [71]. Immunosuppression entails the risk of reactivation of the acute form of CD, and therefore, prophylactic antiparasitic treatment may be indicated for CD-positive transplant recipients [71, 72].

## Treatment of gastrointestinal complications

Gastrointestinal manifestations of chronic CD, most commonly megacolon and megaesophagus, are rarely observed in Mexican and Central American patients, but impact ≈15% of patients with *T. cruzi* infection from Brazil, Argentina, Bolivia, Chile, and Paraguay (i.e., the Southern Cone). Of these, 30% exhibit both cardiac and digestive complications. In patients originating from these countries with confirmed *T. cruzi* infection, several signs and symptoms could point to gastrointestinal involvement of CD (for a complete list, see [73]). Dysphagia and regurgitation could be indicative of esophageal involvement, while volvulus, constipation, or irregular bowel movements can result from colonic damage [73]. In cases where there is clinical suspicion of gastrointestinal complications from CD, a barium enema and radiological study are recommended. Early-stage gastrointestinal manifestations are not necessarily a contraindication for etiological treatment [73], but more advanced cases of megaesophagus or megacolon may require surgical correction before any contemplation of antitrypanosomal treatment [20].

## State of clinical research

Development of safer, more effective drugs for etiological treatment of CD is a top priority. Recent clinical trials of posaconazole and ravuconazole showed these agents were initially effective at clearing the parasite, but the effect was not sustained at 12-month follow-up [38–40]. Because benznidazole exhibited superior efficacy in these studies, attention shifted toward improving the dosage regimen of this drug. Evidence emerged that lower doses of benznidazole might still be effective while improving the side effect profile [74, 75]. MULTIBENZ, a clinical trial in Spain, is evaluating efficacy of lower doses of benznidazole, while BENDITA, a proof-of-concept study in Bolivia, is assessing effectiveness of lower doses and durations of benznidazole as a monotherapy and in combination with ravuconazole. Another proof-of-concept study just underway is gauging antitrypanosomal activity of fexinidazole, which was recently proven safe and effective against human African trypanosomiasis [76].

A wide range of research initiatives are focusing on the identification of biomarkers, both to help determine which patients are at risk of progression to chronic symptoms and to measure the effectiveness of antitrypanosomal therapy [77–79]. Finally, development of new diagnostic tools with high specificity and sensitivity, consistent in different patient populations and *T. cruzi* genetic strains, will greatly facilitate widespread screening [80].

## Conclusions

CD is a life-threatening, severely underdiagnosed parasitic infection. Patients who have spent significant time in rural areas of Latin America or who report being bitten by or in contact with triatomines will benefit from screening for *T. cruzi* infection. Antitrypanosomal therapy using benznidazole or nifurtimox is highly effective for treating the acute phase of the disease, congenitally transmitted cases in infants, and reactivation in immunosuppressed patients. Additionally, treatment with these antiparasitic drugs prevents future congenital transmission. Efficacy in the chronic indeterminate phase exceeds 60% in children. This rate is lower in adults, yet this could be due to the difficulty of measuring seroreversion in chronic patients. However, antitrypanosomal therapy improves clinical outcomes for patients in the chronic indeterminate phase by slowing or avoiding the progression to the advanced chronic form of the disease, resulting in improved outcomes in terms of mortality and morbidity, and should therefore be offered to patients in the absence of contraindications. Although benznidazole and nifurtimox, the only available drugs with proven efficacy against *T. cruzi*, may cause side effects in a substantial portion of patients, these can be successfully managed through a program of close monitoring. CCM is often accompanied by severe damage to the conduction system. Baseline abnormalities on ECG or echocardiogram should trigger an immediate referral to a cardiologist and further evaluation with a 24-h Holter and chest X-ray. A defibrillator and heart failure treatment may be required. Digestive manifestations may affect another subset of patients, especially those born in the Southern Cone of South America. Early screening, diagnosis and treatment is highly cost-saving and critical to preventing the long-term complications of CD. This should be initiated in primary care settings.

## References

[1] Chagas disease in Latin America: an epidemiological update based on 2010 estimates World Health Organization; 2015. Contract No.: 90. 25671846

[2] Manne-Goehler J, Umeh CA, Montgomery SP, Wirtz VJ (2016). Estimating the burden of Chagas disease in the United States. PLoS Negl Trop Dis.

[3] Basile L, Jansa J, Salamanca D, Bartoloni A, Selxas J, Van Gool T (2011). Chagas disease in European countries: the challenge of a surveillance system. Eur Secur.

[4] Garcia MN, Aguilar D, Gorchakov R, Rossmann SN, Montgomery SP, Rivera H (2015). Evidence of autochthonous Chagas disease in southeastern Texas. Am J Trop Med Hyg.

[5] Hernandez S, Flores CA, Viana GM, Sanchez DR, Traina MI, Meymandi SK (2016). Autochthonous transmission of Trypanosoma cruzi in Southern California. Open Forum Infect Dis.

[6] Howard EJ, Xiong X, Carlier Y, Sosa-Estani S, Buekens P (2014). Frequency of the congenital transmission of Trypanosoma cruzi: a systematic review and meta-analysis. BJOG Int J Obstet Gynaecol.

[7] Carlier Y, Torrico F, Sosa-Estani S, Russomando G, Luquetti A, Freilij H, Albajar Vinas P (2011). Congenital Chagas disease: recommendations for diagnosis, treatment and control of newborns, siblings and pregnant women. PLoS Negl Trop Dis.

[8] Rassi A, Rassi A, Marin-Neto JA (2010). Chagas disease. Lancet.

[9] Ribeiro AL, Nunes MP, Teixeira MM, Rocha MO (2012). Diagnosis and management of Chagas disease and cardiomyopathy. Nat Rev Cardiol.

[10] Rassi A, Marin JA, Rassi A (2017). Chronic Chagas cardiomyopathy: a review of the main pathogenic mechanisms and the efficacy of aetiological treatment following the BENznidazole Evaluation for Interrupting Trypanosomiasis (BENEFIT) trial. Mem Inst Oswaldo Cruz.

[11] Viotti R, Alarcón de Noya B, Araujo-Jorge T, Grijalva MJ, Guhl F, López MC, Ramsey JM, Ribeiro I, Schijman AG, Sosa-Estani S, Torrico F, Gascon J, Latin American Network for Chagas Disease, NHEPACHA (2014). Towards a paradigm shift in the treatment of chronic Chagas disease. Antimicrob Agents Chemother.

[12] Zingales B, Miles MA, Campbell DA, Tibayrenc M, Macedo AM, Teixeira MM, et al. The revised Trypanosoma cruzi subspecific nomenclature: rationale, epidemiological relevance and research applications. Infect Genet Evol. 2012;12(2):240–253 10.1016/j.meegid.2011.12.009 22226704

[13] Zingales B, Miles MA, Moraes CB, Luquetti A, Guhl F, Schijman AG, Ribeiro I, Drugs for Neglected Disease Initiative, Chagas Clinical Research Platform Meeting (2014). Drug discovery for Chagas disease should consider Trypanosoma cruzi strain diversity. Mem Inst Oswaldo Cruz.

[14] Pittella JEH (2009). Central nervous system involvement in Chagas disease: a hundred-year-old history. Trans R Soc Trop Med Hyg.

[15] Global Health Estimates (GHE) (2014). 2014 summary tables.

[16] Lee BY, Bacon KM, Bottazzi ME, Hotez PJ (2013). Global economic burden of Chagas disease: a computational simulation model. Lancet Infect Dis.

[17] Requena-Mendez A, Bussion S, Aldasoro E, Jackson Y, Angheben A, Moore D (2017). Cost-effectiveness of Chagas disease screening in Latin American migrants at primary health-care centres in Europe: a Markov model analysis. Lancet Glob Health.

[18] Manne-Goehler J, Reich MR, Wirtz VJ (2015). Access to care for Chagas disease in the United States: a health systems analysis. Am J Trop Med Hyg.

[19] Stimpert KK, Montgomery SP (2010). Physician awareness of Chagas disease, USA. Emerg Infect Dis.

[20] Bern C, Montgomery SP, Herwaldt BL, Rassi A, Marin-Neto JA, Dantas RO, Maguire JH, Acquatella H, Morillo C, Kirchhoff LV, Gilman RH, Reyes PA, Salvatella R, Moore AC (2007). Evaluation and treatment of Chagas disease in the United States: a systematic review. JAMA.

[21] Meymandi SK, Forsyth CJ, Soverow J, Hernandez S, Sanchez D, Montgomery SP, Traina M (2017). Prevalence of Chagas disease in the Latin American–born population of Los Angeles. Clin Infect Dis.

[22] Control de la Enfermedad de Chagas. World Health Organization; 2002.

[23] Otani MM, Vinelli E, Kirchhoff LV, Del Pozo A, Sands A, Vercauteren G (2009). WHO comparative evaluation of serologic assays for Chagas disease. Transfusion.

[24] Dias JCP, Ramos AN Jr, Gontijo ED, Luquetti A, Shikanai-Yasuda MA, Coura JR, et al. Second Brazilian consensus on Chagas disease, 2015. Rev Soc Bras Med Trop. 2016;49:3–60. 10.1590/0037-8682-0505-2016 27982292

[25] Verani JR, Seitz A, Gilman RH, LaFuente C, Galdos-Cardenas G, Kawai V, de LaFuente E, Ferrufino L, Bowman NM, Pinedo-Cancino V, Levy MZ, Steurer F, Todd CW, Kirchhoff LV, Cabrera L, Verastegui M, Bern C (2009). Geographic variation in the sensitivity of recombinant antigen-based rapid tests for chronic Trypanosoma cruzi infection. Am J Trop Med Hyg.

[26] Zingales B. Trypanosoma cruzi genetic diversity: something new for something known about Chagas disease manifestations, serodiagnosis and drug sensitivity. Acta Trop. 2017. 10.1016/j.actatropica.2017.09.017 28941731

[27] Martin DL, Marks M, Galdos-Cardenas G, Gilman RH, Goodhew B, Ferrufino L (2014). Regional variation in the correlation of antibody and T-cell responses to Trypanosoma cruzi. Am J Trop Med Hyg.

[28] Sperandio da Silva GM, Mediano MFF, Hasslocher-Moreno AM, Holanda MT, Silvestre de Sousa A, Sangenis LHC (2017). Benznidazole treatment safety: the Medecins Sans Frontieres experience in a large cohort of Bolivian patients with Chagas’ disease. J Antimicrob Chemother.

[29] Viotti R, Vigliano C, Lococo B, Alvarez MG, Petti M, Bertocchi G, Armenti A (2009). Side effects of benznidazole as treatment in chronic Chagas disease: fears and realities. Expert Rev Anti-Infect Ther.

[30] Alonso-Vega C, Billot C, Torrico F (2013). Achievements and challenges upon the implementation of a program for national control of congenital Chagas in Bolivia: results 2004–2009. PLoS Negl Trop Dis.

[31] Cancado JR (2002). Long term evaluation of etiological treatment of chagas disease with benznidazole. Rev Inst Med Trop Sao Paulo.

[32] Sosa Estani S, Segura EL, Ruiz AM, Velazquez E, Porcel BM, Yampotis C (1998). Efficacy of chemotherapy with benznidazole in children in the indeterminate phase of Chagas’ disease. Am J Trop Med Hyg.

[33] Fabbro DL, Streiger ML, Arias ED, Bizai ML, del Barco M, Amicone NA (2007). Trypanocide treatment among adults with chronic Chagas disease living in Santa Fe city (Argentina), over a mean follow-up of 21 years: parasitological, serological and clinical evolution. Rev Soc Bras Med Trop.

[34] Pérez-Molina JA, Pérez-Ayala A, Moreno S, Fernández-González MC, Zamora J, López-Velez R (2009). Use of benznidazole to treat chronic Chagas’ disease: a systematic review with a meta-analysis. J Antimicrob Chemother.

[35] Sosa-Estani S, Colantonio L, Segura EL (2012). Therapy of Chagas disease: implications for levels of prevention. J Trop Med.

[36] Morillo CA, Marin-Neto JA, Avezum A, Sosa-Estani S, Rassi AJ, Rosas F (2015). Randomized trial of benznidazole for chronic Chagas’ cardiomyopathy. N Engl J Med.

[37] Viotti R, Vigliano C, Lococo B, Bertocchi G, Petti M, Alvarez MG, Postan M, Armenti A (2006). Long-term cardiac outcomes of treating chronic Chagas disease with benznidazole versus no treatment: a nonrandomized trial. Ann Intern Med.

[38] Molina I, Gómez i, Prat J, Salvador F, Treviño B, Sulleiro E, Serre N (2014). Randomized trial of posaconazole and benznidazole for chronic Chagas disease. N Engl J Med.

[39] Torrico F, Gascon J, Ortiz L, Alonso-Vega C, Pinazo MJ, Schijman A, Almeida IC, Alves F, Strub-Wourgaft N, Ribeiro I, Santina G, Blum B, Correia E, Garcia-Bournisen F, Vaillant M, Morales JR, Pinto Rocha JJ, Rojas Delgadillo G, Magne Anzoleaga HR, Mendoza N, Quechover RC, Caballero MYE, Lozano Beltran DF, Zalabar AM, Rojas Panozo L, Palacios Lopez A, Torrico Terceros D, Fernandez Galvez VA, Cardozo L, Cuellar G, Vasco Arenas RN, Gonzales I, Hoyos Delfin CF, Garcia L, Parrado R, de la Barra A, Montano N, Villarroel S, Duffy T, Bisio M, Ramirez JC, Duncanson F, Everson M, Daniels A, Asada M, Cox E, Wesche D, Diderichsen PM, Marques AF, Izquierdo L, Sender SS, Reverter JC, Morales M, Jimenez W (2018). Treatment of adult chronic indeterminate Chagas disease with benznidazole and three E1224 dosing regimens: a proof-of-concept, randomised, placebo-controlled trial. Lancet Infect Dis.

[40] Morillo CA, Waskin H, Sosa-Estani S, Del Carmen Bangher M, Cuneo C, Milesi R (2017). Benznidazole and posaconazole in eliminating parasites in asymptomatic T. cruzi carriers: the STOP-CHAGAS trial. J Am Coll Cardiol.

[41] Sosa-Estani S, Cura E, Velazquez E, Yampotis C, Segura EL (2009). Etiological treatment of young women infected with Trypanosoma cruzi, and prevention of congenital transmission. Rev Soc Bras Med Trop.

[42] Fabbro DL, Danesi E, Olivera V, Codebó MO, Denner S, Heredia C, Streiger M, Sosa-Estani S (2014). Trypanocide treatment of women infected with Trypanosoma cruzi and its effect on preventing congenital Chagas. PLoS Negl Trop Dis.

[43] Moscatelli G, Moroni S, García-Bournissen F, Ballering G, Bisio M, Freilij H, Altcheh J (2015). Prevention of congenital Chagas through treatment of girls and women of childbearing age. Mem Inst Oswaldo Cruz.

[44] Pautas para la atención al paciente infectado con Trypanosoma cruzi (Enfermedad de Chagas). In: Chabén” INdPDMF, editor. Buenos Aires: Ministerio de Salud; 2015.

[45] Garcia-Bournissen F, Altcheh J, Panchaud A, Ito S (2010). Is use of nifurtimox for the treatment of Chagas disease compatible with breast feeding? A population pharmacokinetics analysis. Arch Dis Child.

[46] Garcia-Bournissen F, Moroni S, Marson ME, Moscatelli G, Mastrantonio G, Bisio M (2015). Limited infant exposure to benznidazole through breast milk during maternal treatment for Chagas disease. Arch Dis Child.

[47] Roca Saumell C, Soriano-Arandes A, Solsona Díaz L, Gascón Brustenga J (2015). Documento de consenso sobre el abordaje de la enfermedad de Chagas en atención primaria de salud de áreas no endémicas. Aten Primaria.

[48] Kuschnir E, Sgammini H, Castro R, Evequoz C, Ledesma R, Brunetto J (1985). Evaluation of cardiac function by radioisotopic angiography, in patients with chronic Chagas cardiopathy. Arq Bras Cardiol.

[49] Pinazo M-J, Muñoz J, Posada E, López-Chejade P, Gállego M, Ayala E (2010). Tolerance of benznidazole in treatment of Chagas’ disease in adults. Antimicrob Agents Chemother.

[50] Miller DA, Hernandez S, Rodriguez De Armas L, Eells SJ, Traina MM, Miller LG (2015). Tolerance of benznidazole in a United States Chagas disease clinic. Clin Infect Dis.

[51] Hasslocher-Moreno AM, do Brasil PE, de Sousa AS, Xavier SS, Chambela MC, Sperandio da Silva GM (2012). Safety of benznidazole use in the treatment of chronic Chagas’ disease. J Antimicrob Chemother.

[52] Olivera MJ, Cucunubá ZM, Valencia-Hernández CA, Herazo R, Agreda-Rudenko D, Flórez C, Duque S, Nicholls RS (2017). Risk factors for treatment interruption and severe adverse effects to benznidazole in adult patients with Chagas disease. PLoS One.

[53] Molina I, Salvador F, Sánchez-Montalvá A, Treviño B, Serre N, Sao Avilés A, Almirante B (2015). Toxic profile of benznidazole in patients with chronic Chagas disease: risk factors and comparison of the product from two different manufacturers. Antimicrob Agents Chemother.

[54] Hernandez S, Forsyth CJ, Flores Castro JA, Lemus Valle O, Marquez Lizama G, Sermeno C, et al. Impact of daily dosage limits on frequency and severity of side effects in adult Chagas disease patients treated with benznidazole in a U.S. Clinic. American Society of Tropical Medicine and Hygiene Annual Meeting November 8; Baltimore, 2017.

[55] Jackson Y, Alirol E, Getaz L, Wolff H, Combescure C, Chappuis F (2010). Tolerance and safety of nifurtimox in patients with chronic Chagas disease. Clin Infect Dis.

[56] Bianchi F, Cucunubá Z, Guhl F, González NL, Freilij H, Nicholls RS, Ramírez JD, Montilla M, Flórez AC, Rosas F, Saavedra V, Silva N (2015). Follow-up of an asymptomatic Chagas disease population of children after treatment with nifurtimox (Lampit) in a sylvatic endemic transmission area of Colombia. PLoS Negl Trop Dis.

[57] Forsyth CJ, Hernandez S, Olmedo W, Abuhamidah A, Traina MI, Sanchez DR, Soverow J, Meymandi SK (2016). Safety profile of nifurtimox for treatment of Chagas disease in the United States. Clin Infect Dis.

[58] Perez-Molina JA, Sojo-Dorado J, Norman F, Monge-Maillo B, Diaz-Menendez M, Albajar-Vinas P (2013). Nifurtimox therapy for Chagas disease does not cause hypersensitivity reactions in patients with such previous adverse reactions during benznidazole treatment. Acta Trop.

[59] Nunes MCP, Barbosa MM, Ribeiro ALP, Fenelon LMA, Rocha MOC (2010). Factores predictivos de la mortalidad en pacientes con miocardiopatía dilatada: importancia de la enfermedad de Chagas como etiología. Rev Esp Cardiol.

[60] Traina MI, Sanchez DR, Hernandez S, Bradfield JS, Labedi MR, Ngab TA, Steurer F, Montgomery SP, Meymandi SK (2015). Prevalence and impact of Chagas disease among Latin American immigrants with nonischemic cardiomyopathy in Los Angeles, California. Circ Heart Fail.

[61] Rassi AJ, Rassi A, Little WC, Xavier SS, Rassi SG, Rassi AG (2006). Development and validation of a risk score for predicting death in Chagas’ heart disease. N Engl J Med.

[62] Andrade JP, Marin-Neto JA, Paola AAV, Vilas-Boas F, Oliveira GMM, Bacal F (2011). I Diretriz Latino-Americana para o Diagnóstico e Tratamento da Cardiopatia Chagásica. Arq Bras Cardiol.

[63] Yancy CW, Jessup M, Bozkurt B, Butler J, Casey DE, Colvin MM, et al. 2017 ACC/AHA/HFSA Focused Update of the 2013 ACCF/AHA Guideline for the Management of Heart Failure: a report of the American College of Cardiology/American Heart Association Task Force on Clinical Practice Guidelines and the Heart Failure Society of America. Circulation. 2017.

[64] Costa R, Rassi A, MIdP L (2004). Estudo clínico e epidemiológico de pacientes submetidos a implante de marcapasso cardíaco artificial permanente: comparação dos portadores da doença de Chagas com os de doenças degenerativas do sistema de condução. Braz J Cardiovasc Surg.

[65] Park S, Sanchez DR, Traina MI, Bradfield JS, Hernandez S, Ufion AJA (2017). The prevalence of Chagas disease among Latin American immigrants with pacemakers in Los Angeles, California. Am J Trop Med Hyg.

[66] Traina M, Meymandi S, Bradfield JS (2016). Heart failure secondary to Chagas disease: an emerging problem in non-endemic areas. Curr Heart Fail Rep.

[67] Martinelli M, Rassi A, Marin-Neto JA, de Paola AA, Berwanger O, Scanavacca MI (2013). CHronic use of Amiodarone aGAinSt Implantable cardioverter-defibrillator therapy for primary prevention of death in patients with Chagas cardiomyopathy study: rationale and design of a randomized clinical trial. Am Heart J.

[68] Botoni FA, Poole-Wilson PA, Ribeiro ALP, Okonko DO, Oliveira BMR, Pinto AS, et al. A randomized trial of carvedilol after renin-angiotensin system inhibition in chronic Chagas cardiomyopathy. Am Heart J. 153(4):544.e1–.e8. 10.1016/j.ahj.2006.12.017 17383291

[69] Issa VS, Amaral AF, Cruz FD, Ferreira SM, Guimaraes GV, Chizzola PR (2010). Beta-blocker therapy and mortality of patients with Chagas cardiomyopathy: a subanalysis of the REMADHE prospective trial. Circ Heart Fail.

[70] Bestetti RB, Theodoropoulos TAD (2009). A systematic review of studies on heart transplantation for patients with end-stage Chagas heart disease. J Card Fail.

[71] Benatti RD, Oliveira GH, Bacal F. Heart transplantation for Chagas cardiomyopathy. J Heart Lung Transplant. 36(6):597–603. 10.1016/j.healun.2017.02.006 28284779

[72] Kransdorf EP, Czer LSC, Luthringer DJ, Patel JK, Montgomery SP, Velleca A, Mirocha J, Zakowski PC, Zabner R, Gaultier CR, Qvarnstrom Y, Benedict T, Steurer F, Bosserman E, Paddock CD, Rafiei M, Kobashigawa JA (2013). Heart transplantation for Chagas cardiomyopathy in the United States. Am J Transplant.

[73] Pinazo MJ, Cañas E, Elizalde JI, García M, Gascón J, Gimeno F, Gomez J, Guhl F, Ortiz V, Posada EJ, Puente S, Rezende J, Salas J, Saravia J, Torrico F, Torrus D, Treviño B (2010). Diagnosis, management and treatment of chronic Chagas’ gastrointestinal disease in areas where Trypanosoma cruzi infection is not endemic. Gastroenterol Hepatol.

[74] Bermudez J, Davies C, Simonazzi A, Pablo Real J, Palma S (2016). Current drug therapy and pharmaceutical challenges for Chagas disease. Acta Trop.

[75] Álvarez MG, Hernández Y, Bertocchi G, Fernández M, Lococo B, Ramírez JC, Cura C, Albizu CL, Schijman A, Abril M, Sosa-Estani S, Viotti R (2016). New scheme of intermittent benznidazole administration in patients chronically infected with Trypanosoma cruzi: a pilot short-term follow-up study with adult patients. Antimicrob Agents Chemother.

[76] Mesu VKBK, Kalonji WM, Bardonneau C, Mordt OV, Blesson S, Simon F, et al. Oral fexinidazole for late-stage African <em>Trypanosoma brucei gambiense</em> trypanosomiasis: a pivotal multicentre, randomised, non-inferiority trial. Lancet. 2017. 10.1016/S0140-6736(17)32758-7 29113731

[77] Requena-Méndez A, López MC, Angheben A, Izquierdo L, Ribeiro I, Pinazo M-J, Gascon J, Muñoz J (2013). Evaluating Chagas disease progression and cure through blood-derived biomarkers: a systematic review. Expert Rev Anti-Infect Ther.

[78] Pinazo M-J, Thomas MC, Bua J, Perrone A, Schijman A-G, Viotti R-J, Ramsey JM, Ribeiro I, Sosa-Estani S, López MC, Gascon J (2014). Biological markers for evaluating therapeutic efficacy in Chagas disease, a systematic review. Expert Rev Anti-Infect Ther.

[79] Pinazo M-J, Thomas M-C, Bustamante J, de Almeida IC, Lopez M-C, Gascon J (2015). Biomarkers of therapeutic responses in chronic Chagas disease: state of the art and future perspectives. Mem Inst Oswaldo Cruz.

[80] Balouz V, Agüero F, Buscaglia CA (2017). Chagas disease diagnostic applications: present knowledge and future steps. Adv Parasitol.

